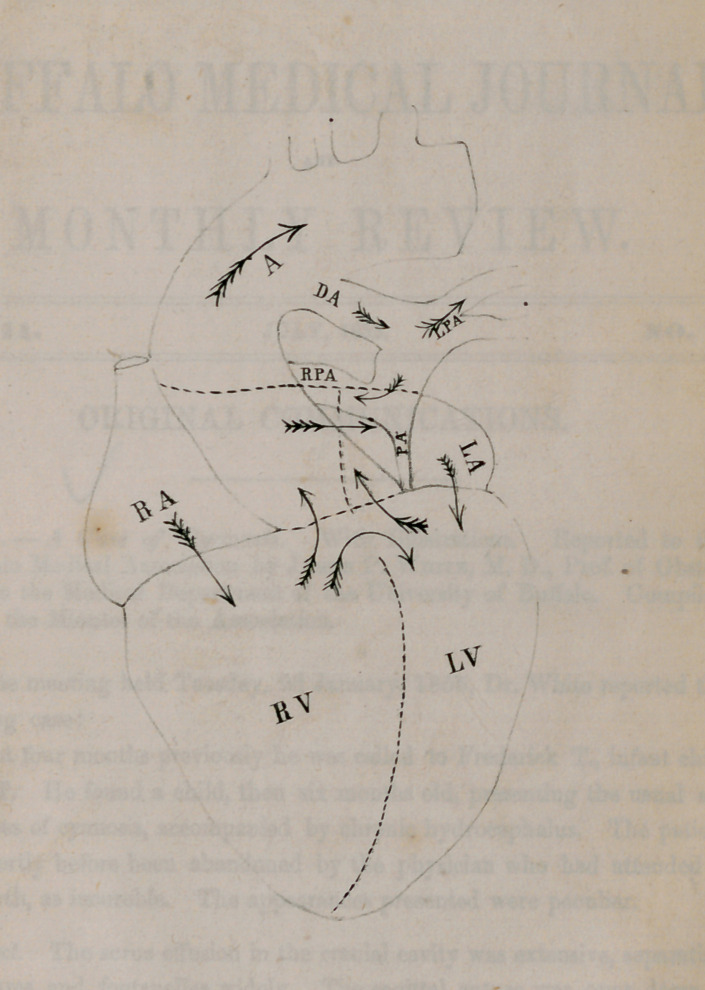# A Case of Cyanosis

**Published:** 1855-07

**Authors:** James P. White

**Affiliations:** Prof. of Obstetrics in the Medical Department of the University of Buffalo


BUFFALO MEDICAL JOURNAL

AND

MONTHLY REVIEW.

VOL. 11. JULY, 1855. NO. 2.

### ORIGINAL COMMUNICATIONS.

ART. I.—*A Case of Cyanosis.* With Illustrations. Reported to the Buffalo Medical Association by JAMES P. WHITE, M. D., Prof. of Obstetrics in the Medical Department of the University of Buffalo. Compiled from the Minutes of the Association.

At the meeting held Tuesday, 2d January, 1855, Dr. White reported the following case:

About four months previously he was called to Frederick T., infant child of Mr. T. He found a child, then six months old, presenting the usual appearances of cyanosis, accompanied by chronic hydrocephalus. The patient had shortly before been abandoned by the physician who had attended it from birth, as incurable. The appearances presented were peculiar.

*Aspect.* The serus effusion in the cranial cavity was extensive, separating the sutures and fontanelles widely. The sagittal suture was open down to the ossa nasi, and these seemed pushed apart from each other, so as to give a peculiar flattened appearance to the nose. The form was emaciated, the entire weight of the patient, at six months old, being about the same as at birth, 8½ pounds. The complexion was cyanotic. The general blueness which overspread the surface, was at all times clearly perceptible, but in the presence of fatigue, excitement, or a fit of coughing, it became intensely blue. In its ordinary aspect the blueness amounted to a deep purple lividity about the lips, inside of the mouth, and under the nails.

*Respiration and Circulation.* The act of breathing was irregular and difficult, the expansion of the chest imperfect, and abdominal breathing and puerile bronchial sounds were both more marked than usual. The action of the heart was rapid and tumultuous, so much so as to render impossible any analysis of its sounds. The surface, particularly of the extremities, was cold.

Before describing the treatment adopted, Dr. White spoke at some length of the pathology of cyanosis. The usual opinion that it depended on non-closure of the foramen ovale was not confirmed by probability, or the results of *post-mortem* examinations. A patulous condition of the foramen ovale was not enough to account for the symptoms. The intermixture of venous and arterial blood through that opening could not be large, and cases had been known where this foramen was found open on *post-mortem* examination, and no cyanosis had existed before death. Other causes might produce the blueness of surface. Anything causing imperfect hematosis would do so. Again, the non-closure of the foramen ovale was not the only means of admixture of the two kinds of blood. Sometimes the opening was interventricular; sometimes the pulmonary artery was too small or impervious; and sometimes the ductus arteriosus remained open. Many cases of this sort had been recorded.

*Treatment.* The hydrocephalic condition Dr. White considered as consequent upon the imperfect aeration of the blood, and therefore it did not require any special treatment. The plan pursued was supporting. Iodide of potassium, cod liver oil, and brandy were given. The diet was nutritious, consisting of fat food, and as concentrated as possible. Exercise by carrying in the out-door air, was insisted upon, and the heat of the surface was promoted by warm clothing. Among other means used was that recommended by Prof. Meigs, of obliging the patient to lie upon the right side. This was entirely ineffectual, no result, either good or bad, being obtained.

Under this management the child had improved to a very marked degree during the four months it had been under treatment. Its weight is now 13½ pounds; the cyanosis is less marked; the respiration more even; and the size of the head considerably diminished. The sutures now approximate each other, and the fontanelles are less protuberant.

Recently it has suffered from an acute catarrhal attack, by which the respiration was very much impeded, from the secretion of mucus, etc. During this the cyanosis was very profound, the lips and inside of mouth being of a deep Isabella grape color, and death from asphyxia seemed threatened. It was, however, speedily relieved by expectorants, combined with diffusible stimulus.

At the meeting of the association held Tuesday, April 3, Dr. White reported that the case of cyanosis reported at the January meeting, had terminated fatally a few days before. During the three months which had elapsed since the last report, the case had gone on favorably. The weight of the child at thirteen months was 16½ pounds, being 3 pounds more than reported in January. The aspect of the child had correspondingly improved. The hydrocephalic condition had almost entirely disappeared, and the cyanosis was less marked than formerly.

On Sunday the 24th of March, he had eaten an unusually hearty breakfast, and had been allowed to partake freely of corn cake, with the idea of obviating a degree of costiveness present at the time. The parents left it for church without the usual direction to the nurse to send for the doctor if anything unusual should occur — so much better than usual did it appear. They were called out during service, and it died soon after their return, in a convulsion of some kind. No unfavorable appearance had been noticed, with the exception of an unusual fullness of the fontanelle.

A *post-mortem* examination was made on Monday, the 25th. Present, Drs. White, Hunt, and Howell.

*Aspect.* Considerable rigor mortis. The cyanotic condition quite evident in the deep color of the lips, and the blue stain under the nails.

Only the chest was examined, some difficulty having been met in obtaining the consent of the friends. The thymus gland was not particularly noticed, but it is certain that there was no unusual enlargement of it. The lungs were carnified in scattered portions, so much so as to present unusual solidity. Here and there they presented the usual spongy, crepitant tissue, but in many portions, particularly about the base, they were so solid that it was evident they had never been fully expanded by air. No evidences of inflammatory action, or of collapse of lung subsequent to expansion, were discovered. The heart was removed and exhibited to the association. The life-size plates which accompany this description, give a very clear idea of the conditions found.

The upper plate on the left hand page, gives a front view of the heart, and a portion of the lung suspended by the trachea. The right side of the heart is much the largest, the comparative size being very accurately shown in the plate. The right auricle is proportionally much larger than usual, while the left side is dwarfed. The weight of the whole organ was 2oz. 6ʒ.

A section made in the left auricle exhibits the foramen ovale in a patulous condition, partially closed by the Eustachian valve. Above, upon the right auricle, is seen the opening of the descending vena-cava.

The lower plate on the left-hand page, gives the posterior view of the heart and great bloodvessels. Only a small portion of the left ventricle is exhibited, while the comparative size of the right auricle and ventricle are very well given. The aorta is seen of an enormous size at its origin, rapidly narrowed where it gives off the ductus arteriosus. To the right of the aorta is seen the pulmonary artery—beginning at the conus arteriosus in a slender impervious cord.

Leaving the aorta just beneath its arch, is seen the ductus arteriosus, large and pervious, breaking into two portions at the distance of eight or nine lines from its origin. One of these branches, forming the left pulmonary artery, proceeds directly on to the lung of that side; the other, curving downward and to the right, passes over to the right lung, making the right pulmonary artery. At the lowest point of its curve, it is continuous with a cul de sac occupying a portion of the obliterated common pulmonary artery. These fœtal branches of the ductus arteriosus form the only channel for the circulation to the lung.

On the opposite page is an ideal diagram of the circulation, which exhibits still another malformation which could not be easily shown in the drawings. The inter-ventricular septum is wanting at its upper portion, and the origin of the aorta is situated immediately over it so as to receive the blood from both ventricles.

The letters upon the diagram, refer to the parts indicated, as follows:

A. Aorta.
D. A. Ductus Arteriosus.
L. P. A. Left Pulmonary Artery.
R. P. A. Right Pulmonary Artery.
P. A. Common Pulmonary Artery.
R. A. Right Auricle.
L. A. Left Auricle.
R. V. Right Ventricle.
L. V. Left Ventricle.

The arrows indicate the course of the circulation. Blood received into the right auricle was transmitted either to the right ventricle or the left ventricle, and blood received into the left auricle from the pulmonary veins, passed either to the corresponding ventricle, or the opposite auricle. At the ventricular systole the whole contents of both ventricles passed into the aorta, and through the ductus arteriosus to the lungs and the system in common.

It will be seen that this is, in effect, a reptilian heart, and that for all purposes of life the heart had but one side, the lung sharing with the entire system in the distribution of blood. Of course the aeration was extremely imperfect, and it is truly surprising that the offices of life could have been carried on for thirteen months in such a condition; and that for the last seven months of life there should have been a constant and steady improvement in weight and in the health of the various functions. All the recognized conditions of cyanosis—the open foramen ovale, the closed pulmonary artery, the open ductus arteriosus, an inter-ventricular communication, and an imperfect expansion of lung—are combined in this case.

## Figures and Tables

**Figure f1:**
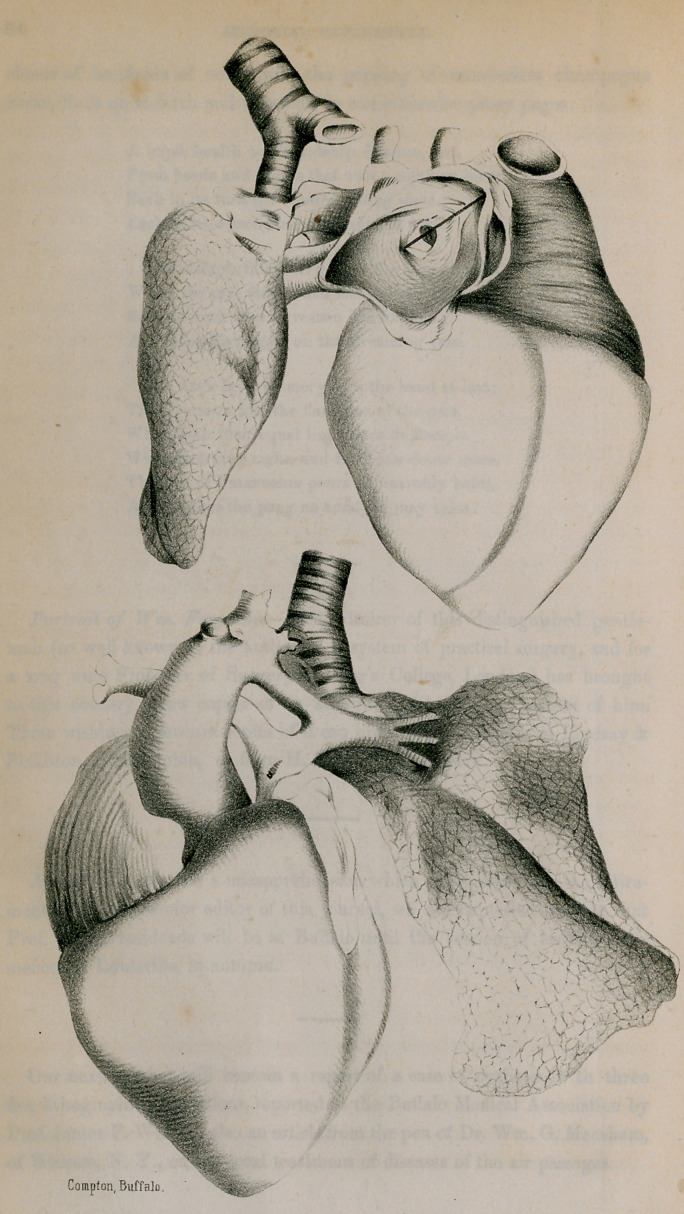


**Figure f2:**